# Microbiota Dysbiosis and the Gut-Brain Axis in Psychiatric Disorders: Molecular Mechanisms, Clinical Evidence, and Therapeutic Perspectives

**DOI:** 10.31083/AP54121

**Published:** 2026-10-22

**Authors:** Giuseppe Marano, Luca Conci, Leila Farsakh, Gianandrea Traversi, Osvaldo Mazza, Valentina Ricozzi, Silvia Rotondaro, Antonella Migliore, Esmeralda Capristo, Gianluca Franceschini, Eleonora Gaetani, Marianna Mazza

**Affiliations:** ^1^Department of Neuroscience, Head-Neck and Chest, Section of Psychiatry, Fondazione Policlinico Universitario Agostino Gemelli IRCCS, 00168 Rome, Italy; ^2^Department of Neuroscience, Section of Psychiatry, Università Cattolica del Sacro Cuore, 00168 Rome, Italy; ^3^Unit of Medical Genetics, Department of Laboratory Medicine, Ospedale Isola Tiberina-Gemelli Isola, 00186 Rome, Italy; ^4^Spine Surgery Department, Bambino Gesù Children’s Hospital IRCCS, 00168 Rome, Italy; ^5^Division of Gynecologic Oncology, Department of Woman and Child Health and Public Health, Fondazione Policlinico Universitario Agostino Gemelli IRCCS, 00168 Rome, Italy; ^6^Department of Medical and Surgical Sciences, Fondazione Policlinico Universitario Agostino Gemelli IRCCS, 00168 Rome, Italy; ^7^Department of Translational Medicine and Surgery, Fondazione Policlinico Universitario Agostino Gemelli IRCCS, Università Cattolica del Sacro Cuore, 00168 Rome, Italy; ^8^Breast Surgery Unit, Department of Woman and Child’s Health and Public Health Sciences, Fondazione Policlinico Universitario A. Gemelli IRCCS, 00168 Rome, Italy; ^9^Department of Medical and Surgical Sciences, Catholic University of the Sacred Heart, 00168 Rome, Italy; ^10^Unit of Internal Medicine, Cristo Re Hospital, 00167 Rome, Italy

**Keywords:** gut-brain axis, gut microbiota, dysbiosis, psychiatric disorders, depression, bipolar disorder, anxiety, schizophrenia, microbiota-targeted interventions

## Abstract

The gut-brain axis provides a biologically plausible framework for understanding the bidirectional interactions among the intestinal microbiota, immune-inflammatory signaling, neuroendocrine regulation, neurotransmitter metabolism, and psychiatric symptoms. Although numerous recent reviews have examined individual psychiatric disorders, microbial composition, or microbiota-targeted therapies, the field still lacks a sufficiently integrated appraisal that clearly distinguishes associative findings from human studies, mechanistic evidence from preclinical research, and preliminary clinical interventions. This structured narrative review synthesizes clinical, translational, and preclinical studies across major depressive disorder, bipolar disorder, anxiety disorders, schizophrenia, and trauma-related conditions. The main literature search covered publications from January 2015 to January 2026; earlier foundational mechanistic studies of key relevance to gut–brain communication were also considered. Recurrent pathways include altered short-chain fatty acid production, tryptophan-kynurenine metabolism, immune-inflammatory activation, hypothalamic-pituitary-adrenal axis dysregulation, neurotransmitter-related signaling, impaired intestinal and blood-brain barrier integrity, and vagus nerve-mediated communication. Human studies predominantly demonstrate associations between microbial signatures and psychiatric phenotypes, symptom severity, inflammatory status, or treatment response, whereas preclinical models provide stronger mechanistic evidence that microbiota manipulation can influence behavioral, neurochemical, immune, and neuroendocrine outcomes. Probiotics, prebiotics, dietary interventions, and fecal microbiota transplantation show therapeutic potential; however, the current evidence is limited by small sample sizes, methodological heterogeneity, insufficient standardization, and a lack of long-term validation. The distinctive contribution of this review is its structured comparison of three levels of evidence—human observational findings, experimental mechanistic studies, and emerging clinical interventions—thereby clarifying what has been consistently replicated, what remains mechanistically plausible, and what is not yet ready for routine application in psychiatric practice.

## Main Points

1. Human studies link gut microbial patterns with psychiatric symptoms, but these associations do not establish causality or direction of effect.

2. Controlled animal studies implicate microbial metabolites, immune and stress pathways, barrier integrity, and vagal signaling in gut–brain communication.

3. Across diagnoses, broad functional pathways appear more consistent than changes in individual microbial taxa; no disorder-specific signature is established.

4. Probiotics, prebiotics, dietary approaches, and fecal microbiota transplantation remain investigational adjuncts, with limited and heterogeneous clinical evidence.

5. Longitudinal, adequately powered studies with standardized microbiome methods and careful control of diet and medication are needed before clinical translation.

## 1. Introduction

### 1.1 Mechanism of the Gut-Brain Axis

Various studies have identified the importance of the link between gut microbiota and mental health, highlighting the importance of mutual communication between the enteric nervous system (ENS) and the central nervous system (CNS) [1,2,3]. An early historical observation of the relationship between emotional states and gastrointestinal function derives from the work of William Beaumont, a surgeon in the United States Army. Between 1822 and 1833, Beaumont conducted physiological observations on Alexis St. Martin, a Canadian voyageur who developed a permanent gastric fistula following an accidental gunshot wound. These investigations allowed direct observation of gastric activity and included descriptions of changes in gastric secretion and mucosal appearance during emotional states [4]. Although these observations predated modern microbiome science, they represent an important historical antecedent of gastrointestinal psychophysiology and the contemporary gut-brain-axis concept.

Understanding these interactions could also lead to new clinical and therapeutic approaches, since modifying gut microbiota composition may influence neural, immune, endocrine, and metabolic processes, although the clinical magnitude and direction of these effects remain uncertain. Indeed, it has been found that there are five communication routes between gut and brain, including the gut-brain’s neural network, neuroendocrine-hypothalamic-pituitary-adrenal (HPA) axis, gut immune system, some neurotransmitters and neural regulators synthesized by gut bacteria, and barrier paths including intestinal mucosal barrier and blood-brain barrier (Fig. 1) [5].

**Fig. 1.**
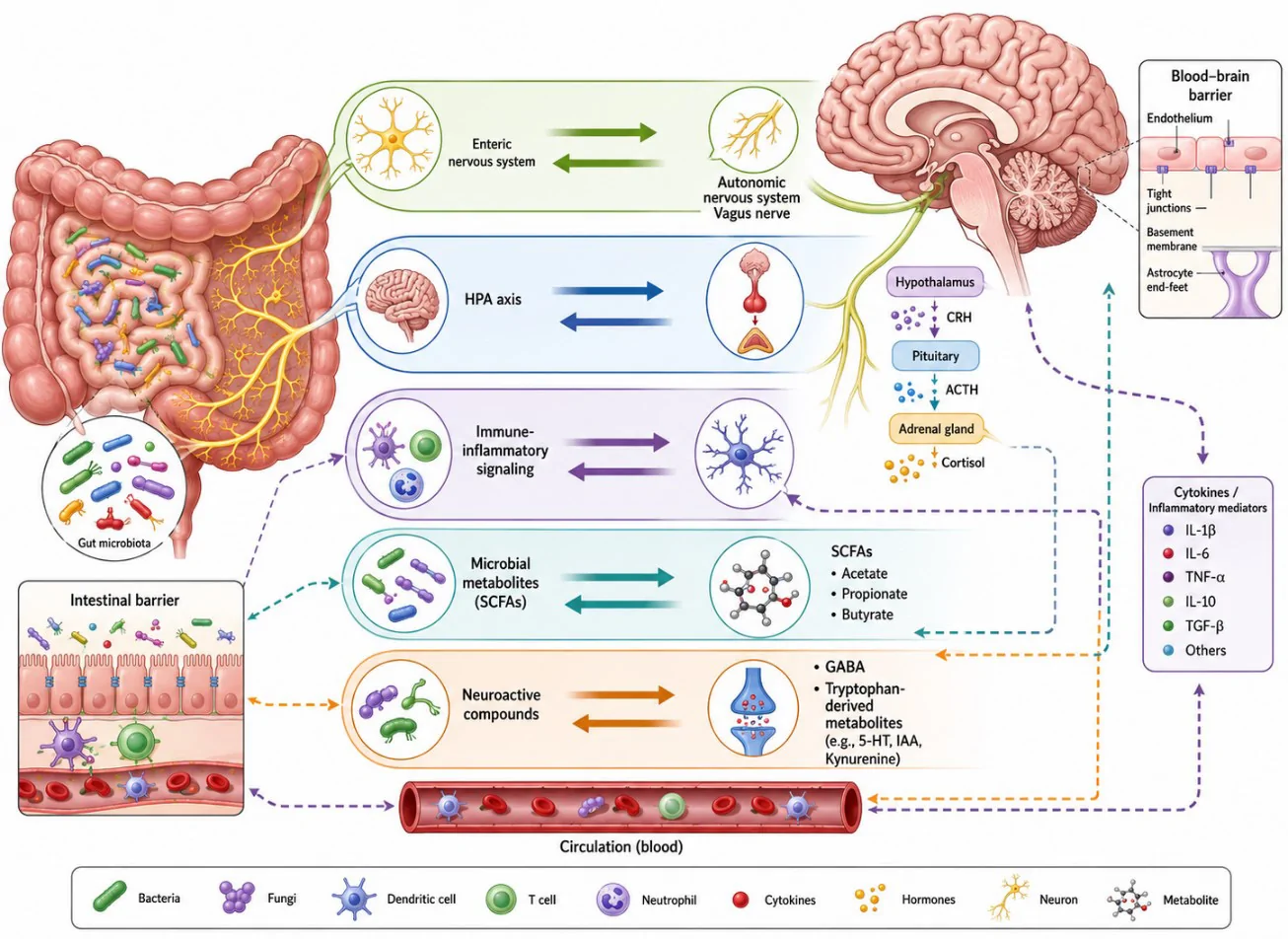
**Schematic representation of bidirectional gut–brain communication**. The figure illustrates the principal pathways connecting the gastrointestinal tract and the brain, including neural signaling through the enteric and autonomic nervous systems, neuroendocrine regulation through the hypothalamic-pituitary-adrenal axis, immune-inflammatory signaling, microbial metabolites and neuroactive compounds, and the intestinal and blood–brain barriers. Short-chain fatty acids are represented as microbial metabolites rather than neurotransmitters. Abbreviations: GABA, γ-aminobutyric acid; HPA, hypothalamic-pituitary-adrenal; SCFAs, short-chain fatty acids. Fig. 1 was created with the assistance of Google Gemini 2.0 (Google; accessed May 2026), under the authors’ guidance and direction. The authors reviewed and edited the final figures and take responsibility for their scientific content.

Enterochromaffin cells in the intestinal mucosa produce most peripheral serotonin, whereas gut microorganisms generate or influence a broad range of neuroactive metabolites. Gut microorganisms and host-microbial metabolic interactions can generate compounds with neuroactive or potentially neurotoxic properties under specific pathological conditions. D-lactate accumulation is primarily associated with conditions such as short-bowel syndrome, intestinal carbohydrate malabsorption, or marked microbial overproduction, where it may contribute to D-lactic acidosis and neurological manifestations. Similarly, elevated ammonia is most clinically relevant in hepatic dysfunction, portosystemic shunting, or severe disturbances of nitrogen metabolism, in which impaired detoxification may lead to neurocognitive effects. These compounds should not be regarded as typical or consistently demonstrated features of microbiota dysbiosis in psychiatric disorders. Rather, they illustrate how gut-derived metabolites may affect brain function when production, absorption, or host clearance becomes pathologically altered. Their direct contribution to common psychiatric phenotypes remains insufficiently established [5].

Preclinical evidence suggests that the gut microbiota may modify stress-related neurotransmitter signaling and neuroendocrine responses. These effects interact with, rather than replace, the direct neurobiological consequences of stress. Experimental evidence suggests that microbiota-related signals may modulate selected enteric serotonergic pathways, including pathways involving 5-HT3 and 5-HT4 receptors, and may influence vagal afferent signaling. However, these effects are model-dependent and do not demonstrate a uniform alteration of enteric receptor systems in human psychiatric disorders [6,7].

On this basis current research has progressively investigated specific neurochemical and receptor-mediated mechanisms through which gut microbiota influences brain function.

### 1.2 Neural Pathways

#### 1.2.1 The Enteric Nervous System (ENS)

The enteric nervous system is an intrinsic neural network embedded within the gastrointestinal wall and organized primarily into the myenteric and submucosal plexuses. Its neuronal circuitry includes intrinsic primary afferent neurons, interneurons, excitatory and inhibitory motor neurons, secretomotor neurons, vasomotor neurons, and enteric glial cells. Through these interconnected circuits, the ENS coordinates gastrointestinal motility, secretion, local blood flow, epithelial transport, barrier function, and interactions with mucosal immune cells. Although enteric circuits can generate and regulate many gastrointestinal responses independently of direct central input, their activity is continuously integrated with sympathetic, parasympathetic, endocrine, immune, and local tissue signals [8,9].

Within the microbiota-gut-brain axis, the ENS should therefore be regarded not as a passive target of microbial activity but as an intrinsic neural interface participating in bidirectional communication among the intestinal environment, the autonomic nervous system, and the central nervous system. Experimental evidence indicates that microbial metabolites and host responses to microbial products may modulate enteric neuronal excitability, glial activity, visceral sensory processing, and selected receptor-mediated pathways. However, effects on specific serotonergic, GABAergic, or other receptor subtypes vary according to the experimental model and should not be interpreted as a uniform or clinically established consequence of dysbiosis. The available evidence consequently supports a modulatory role of microbiota-related signaling within enteric neural circuits, while the magnitude and clinical relevance of these effects in psychiatric populations remain to be established.

#### 1.2.2 Vagal Nerve Signaling

The vagus nerve represents one of the main neural pathways connecting the gut to the brain. A foundational preclinical study published by Bravo et al. in 2011 [10] provided early experimental evidence for the involvement of the vagus nerve in microbiota-brain communication. In mice, administration of *Lactobacillus rhamnosus* JB-1 was associated with changes in anxiety- and depression-like behavior and in central GABA-receptor expression; these effects were abolished following subdiaphragmatic vagotomy [10]. Although this experiment remains influential in establishing vagus-dependent mechanistic plausibility, its findings derive from a specific animal model and should not be interpreted as direct evidence of equivalent effects in humans. Subsequent preclinical research has further supported the role of vagal afferent signaling as one of several pathways through which microbial activity may influence central neurobiological processes [11].

### 1.3 Neurochemical Signaling in the Gut-Brain Axis

Accumulating, predominantly preclinical evidence suggests that gut microbiota may modulate pathways related to central neurotransmission by producing neuroactive molecules. Key neurotransmitters include serotonergic, GABAergic, dopaminergic and glutamatergic pathways which are involved in mood regulation, stress response and cognitive alterations both in animal models and humans [12].

#### 1.3.1 Serotonergic Signaling

One of the most extensively investigated pathways within the gut-brain axis is serotonergic signaling. More than 90% of the body’s serotonin, or 5-hydroxytryptamine (5-HT), is produced in the gastrointestinal tract, predominantly by enterochromaffin cells. Peripheral 5-HT regulates gastrointestinal motility, secretion, vascular tone, platelet function, and local immune signaling. Circulating serotonin does not readily cross the blood-brain barrier and therefore does not directly contribute to central serotonergic neurotransmitter pools. Serotonin in the central nervous system is synthesized locally by serotonergic neurons from circulating tryptophan, which can cross the blood-brain barrier. Accordingly, the gut microbiota may influence central serotonergic function indirectly by modulating tryptophan availability and metabolism, immune-inflammatory signaling, endocrine pathways, vagal afferent activity, and the production of microbial metabolites [13,14].

In addition to influencing tryptophan metabolism and peripheral serotonin production, the gut microbiota may modulate the expression and function of serotonergic receptors and the serotonin transporter in the enteric nervous system. Experimental studies indicate that microbial signals can alter the expression of receptor subtypes such as 5-HT3 and 5-HT4, which contribute to gastrointestinal motility, secretion, and visceral sensory signaling [15]. These findings support an indirect and bidirectional relationship between microbial activity, peripheral serotonergic signaling, and central nervous system function rather than a direct transfer of gut-derived serotonin to the brain.

Foundational germ-free animal studies provided early evidence that the absence of gut microbiota during development may influence stress-related neuroendocrine and neurochemical processes. In a frequently cited 2013 experiment, germ-free mice showed alterations in hippocampal serotonergic signaling and stress-related outcomes compared with conventionally colonized animals [16]. These findings contributed substantially to the mechanistic basis of the microbiota-gut-brain model, but they should be interpreted as developmental and preclinical evidence rather than as direct demonstrations of microbiota-related mechanisms in patients with psychiatric disorders.

#### 1.3.2 GABAergic Signaling

GABA is the main inhibitory neurotransmitter in the CNS, and alterations in GABAergic signaling have been linked to various CNS disorders, including behavioral changes, pain modulation, and sleep disturbances. In the ENS, GABA contributes to intestinal motility, gastric emptying, nociception, and acid secretion. The microbiota also seems to have an impact on circulating GABA levels, as germ-free mice show reduced luminal and serum GABA, although central levels remain unchanged [7].

Consistent with the vagus-dependent effects described above, foundational animal research showed that *Lactobacillus rhamnosus* JB-1 altered central GABA receptor expression and emotional behavior, supporting an interaction between microbial signaling, vagal pathways, and GABAergic circuits [10]. These findings support a central role for GABAergic signaling and other neurotransmitter systems in the regulation of the gut-brain axis.

#### 1.3.3 Dopaminergic Signaling

Dopamine is present in substantial concentrations within the gastrointestinal tract and is produced by enteric neural, epithelial, and microbial pathways. In the gut, dopaminergic signaling participates in the regulation of motility, secretion, epithelial barrier function, and local immune activity. Because peripheral dopamine does not readily cross the blood-brain barrier, its potential influence on central function is thought to occur indirectly through neural, immune, endocrine, and metabolic pathways [17].

Emerging evidence, predominantly from preclinical studies, suggests that gut microbiota may influence peripheral dopamine availability and indirectly modulate central dopaminergic signaling. Genera including *Prevotella*, *Bacteroides*, *Lactobacillus*, and *Bifidobacterium* have been implicated in these interactions through effects on dopamine precursor metabolism and receptor- or transporter-related pathways; however, the mechanisms and strength of evidence differ across taxa and experimental models [17,18].

Additionally, probiotic strains like Lactobacillus rhamnosus and Lactobacillus plantarum have been shown to affect enzymes involved in dopamine synthesis and receptor expression, suggesting mechanisms by which the microbiota can modulate dopaminergic signaling [18].

#### 1.3.4 Glutamatergic Signaling

Glutamate is the primary excitatory neurotransmitter in the CNS, although research has demonstrated its role as a key signaling molecule in the ENS, regulating gut motility, secretion, and visceral sensory signaling. Emerging evidence indicates that microbiota-related metabolites and host responses may modulate glutamatergic pathways in experimental models through production of neuroactive metabolites, modulation of tryptophan metabolism, and regulation of glutamate receptor expression along the gut-brain axis.

Alterations in glutamatergic pathways may also contribute to the pathogenesis of a number of CNS disorders, as well as gastrointestinal diseases [8].

These findings highlight that gut microbiota can influence several neurochemical pathways influencing both CNS and ENS. This intricate regulation of neurotransmission suggests a link between microbial composition and behavioral outcomes, setting the stage for understanding the role of microbiota in mood disorders and related neuropsychiatric conditions. Fig. 2 provides a schematic overview of the key molecular and cellular pathways through which gut microbiota dysbiosis influences brain function.

**Fig. 2.**
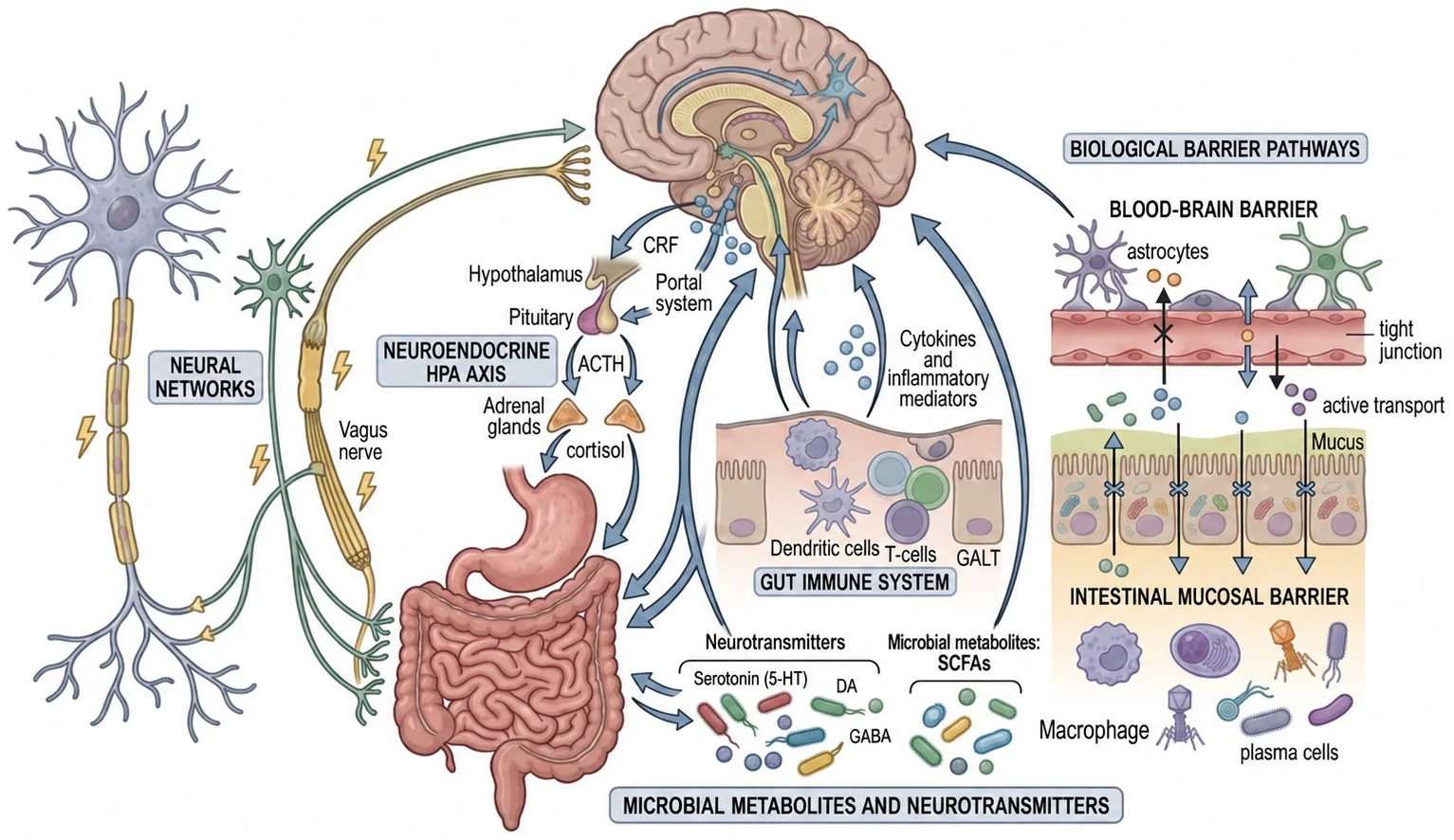
**Biological pathways linking gut microbiota dysbiosis with brain-related outcomes**. Gut microbiota dysbiosis may influence brain function through interacting neural, immune-inflammatory, neuroendocrine, metabolic, and barrier-related pathways. These include altered microbial metabolite production, changes in short-chain fatty acid availability, modulation of tryptophan and neurotransmitter-related pathways, hypothalamic-pituitary-adrenal axis activation, vagal signaling, systemic inflammation, and impaired intestinal and blood-brain barrier integrity. The pathways shown represent biologically plausible mechanisms supported to different degrees by preclinical and human evidence and should not be interpreted as uniformly established causal mechanisms in psychiatric disorders. Abbreviations: 5-HT, 5-hydroxytryptamine (serotonin); ACTH, adrenocorticotropic hormone; CRF, corticotropin-releasing factor; DA, dopamine; GABA, γ-aminobutyric acid; GALT, gut-associated lymphoid tissue; HPA, hypothalamic-pituitary-adrenal; SCFAs, short-chain fatty acids. Fig. 2 was created with the assistance of Google Gemini 2.0 (Google; accessed May 2026), under the authors’ guidance and direction. The authors reviewed and edited the final figures and take responsibility for their scientific content.

### 1.4 Microbiota and Psychiatric Disorders: Rationale for the Review

The growing interest in the gut-brain axis has progressively extended from basic neurogastroenterology to psychiatry. Several psychiatric disorders, including mood disorders, anxiety disorders, schizophrenia, and trauma-related disorders, have been investigated in relation to gut microbiota composition, microbial metabolites, immune-inflammatory activation, neuroendocrine regulation, and neurotransmitter signaling [19,20,21,22,23,24,25].

The interpretation of this literature remains challenging. Human studies are often observational and heterogeneous, while preclinical models provide stronger mechanistic evidence but have limited direct translatability to complex psychiatric phenotypes. Moreover, microbiota-targeted interventions, including probiotics, dietary modulation, prebiotics, and fecal microbiota transplantation (FMT), are promising but remain insufficiently standardized for routine clinical use [26,27].

On this basis, the present review aims to synthesize current evidence on microbiota dysbiosis and gut-brain axis pathways in psychiatric disorders, distinguishing associative clinical findings from mechanistic preclinical evidence and highlighting therapeutic perspectives, methodological limitations, and priorities for future research.

### 1.5 Novelty and Contribution of This Review

Several reviews published between 2023 and 2025 have examined complementary aspects of the gut–brain axis, including mood disorders [2], depressive disorders [20], neurotransmitter-related mechanisms [6], and microbiota-targeted interventions in psychiatric populations [28]. These differing scopes highlight the value of a cross-disorder synthesis that explicitly distinguishes human observational findings, preclinical mechanistic evidence, and clinical intervention outcomes. The present review extends this literature by comparing these domains within a single translational framework and by explicitly separating levels of inference.

The specific contribution of the present review is therefore not the introduction of an entirely new conceptual framework, but the structured comparison of three distinct levels of evidence: associative human studies, mechanistic preclinical investigations, and emerging clinical interventions. By organizing findings according to evidentiary strength and translational relevance, the review aims to clarify which observations are replicated in patients, which mechanisms are primarily supported by experimental models, and which therapeutic approaches remain preliminary.

## 2. Materials and Methods

### 2.1 Study Design

This article is a structured narrative review aimed at providing an integrative and critical synthesis of current evidence on the gut-brain axis in major psychiatric disorders and in experimentally relevant neurobehavioral models. The review was designed to summarize human clinical and observational evidence, translational studies, and preclinical investigations examining microbiota dysbiosis, molecular pathways, and microbiota-targeted interventions. Preclinical studies were considered eligible only when they modeled behavioral, neurochemical, immune, or neuroendocrine processes directly relevant to the psychiatric conditions included in the review. Because the objective was to provide a broad mechanistic and clinical synthesis rather than to answer a narrowly defined intervention question, the review was not conducted as a formal systematic review or meta-analysis. Accordingly, the review was not conducted or reported as a formal PRISMA-compliant systematic review, and no quantitative meta-analysis or formal study-level risk-of-bias assessment was performed. Nevertheless, the PRISMA 2020 flow diagram template was adapted to provide a transparent descriptive account of study identification and selection [29]. The search strategy, eligibility criteria, and data-synthesis procedures were predefined to improve transparency and reproducibility.

### 2.2 Search Strategy and Study Identification

A structured literature search was conducted in PubMed for studies published between January 2015 and January 2026. This interval represented the main publication window; earlier foundational mechanistic studies were also considered when they directly informed key gut–brain pathways, including vagus-dependent signaling and microbial regulation of neuroendocrine and neurochemical processes. The search strategy combined predefined free-text terms related to the gut microbiota, gut-brain axis, psychiatric disorders, and microbiota-targeted interventions using the Boolean operators AND and OR. The complete search string was as follows: (microbiota OR microbiome* OR dysbiosis OR “gut-brain axis” OR psychobiotic* OR “fecal microbiota transplantation” OR probiotic* OR prebiotic*) AND (depression OR “major depressive disorder” OR anxiety OR PTSD OR OCD OR “bipolar disorder” OR schizophrenia OR psychosis OR “psychiatric disorder*”).

### 2.3 Screening and Eligibility Assessment

Titles and abstracts were screened independently by two authors. Full texts of potentially eligible articles were subsequently assessed by the same two reviewers according to the predefined eligibility criteria. Any disagreement concerning eligibility was resolved through discussion and consensus with a third author. Because no duplicate database records were identified, all retrieved records proceeded directly to title and abstract screening.

The main publication window was January 2015 to January 2026. Earlier preclinical studies were eligible when they provided foundational mechanistic evidence directly relevant to the pathways discussed, including Bravo et al. (2011) [10] and Clarke et al. (2013) [16]. These studies were considered alongside the main search-period literature to contextualize subsequent findings.

Eligible studies included human observational studies, longitudinal investigations, clinical trials, and relevant preclinical studies examining the relationship between the gut microbiota, gut-brain-axis pathways, and major depressive disorder, bipolar disorder, anxiety disorders, schizophrenia, trauma-related disorders, or closely related behavioral phenotypes. Preclinical studies were included only when the experimental phenotype and the biological pathways investigated were directly relevant to one or more of these psychiatric domains. Studies focused primarily on unrelated neurological, behavioral, or veterinary outcomes were excluded. Case reports and small case series were retained only when they provided clinically relevant information on emerging interventions, particularly FMT, and were interpreted separately from controlled clinical evidence.

### 2.4 Data Extraction and Synthesis

For each included study, the authors extracted, when available, the study design, population or animal model, psychiatric condition or behavioral phenotype, microbiota-related findings, intervention type, and principal outcomes. Human observational evidence, human interventional evidence, and mechanistic animal evidence were categorized separately to avoid conflating associative and experimental findings.

Because of substantial heterogeneity in populations, microbiota assessment methods, psychiatric outcomes, and intervention protocols, quantitative meta-analysis was not considered appropriate.

Because this work was designed as a structured narrative review and included highly heterogeneous human observational studies, clinical interventions, case-level evidence, and preclinical experiments, no formal study-level risk-of-bias scoring tool was prospectively applied. Methodological quality was therefore considered qualitatively during evidence synthesis, with attention to study design, sample size, control groups, adjustment for confounding, microbiota-assessment methods, follow-up duration, outcome validity, and translational relevance. Findings from uncontrolled studies, case reports, and preclinical models were interpreted separately from controlled human evidence.

## 3. Results

### 3.1 Study Identification and Selection

The PubMed search identified 1126 records. Because the search was conducted in a single bibliographic database and no records from additional database searches were merged, no duplicate database records were identified. Therefore, all 1126 records proceeded to title and abstract screening. Of these, 1020 records were excluded because they did not address psychiatric or behavioral outcomes, gut-brain mechanisms, microbiota composition, or microbiota-targeted interventions.

The full texts of 106 potentially eligible reports were assessed. Sixty-one reports were excluded because of insufficient relevance to the review aims, absence of pertinent psychiatric or behavioral outcomes, inadequate methodological information, or substantial overlap with findings already represented by more comprehensive or methodologically informative studies. A total of 45 studies were included in the final qualitative synthesis: 16 human observational or longitudinal studies, 22 preclinical or translational studies, and 7 human interventional studies (Fig. 3).

**Fig. 3.**
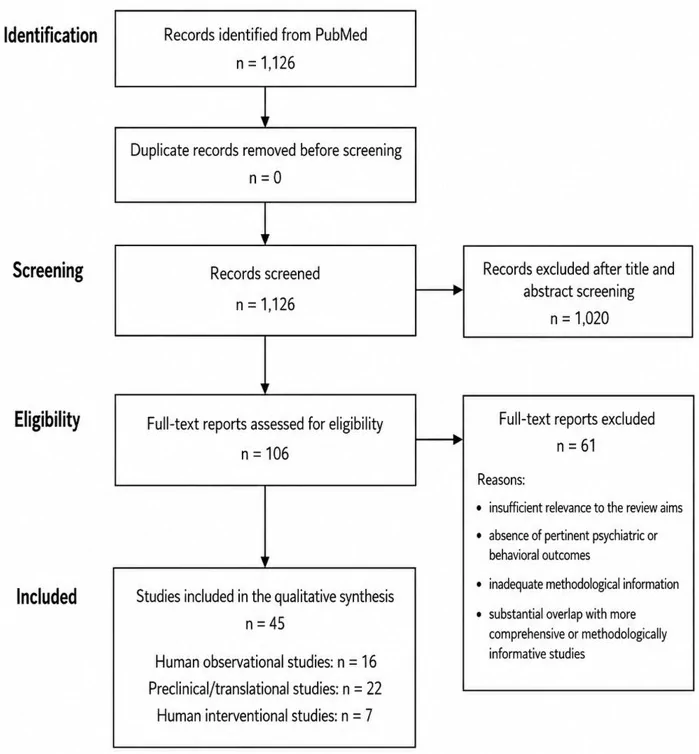
**Study identification and selection process**. Note. The flow diagram was adapted from the PRISMA 2020 flow diagram template and is provided solely to improve transparency in reporting study identification, screening, eligibility assessment, and inclusion. The present article was conducted as a structured narrative review and not as a formal PRISMA-compliant systematic review.

### 3.2 Characteristics of Included Studies

The review included 45 studies comprising human observational and longitudinal investigations, clinical interventions, and preclinical or translational animal experiments. Mood disorders represented the most frequently investigated diagnostic domain, followed by anxiety disorders, schizophrenia, and trauma-related conditions. The number of publications increased during the latter part of the predefined review period, reflecting the rapid development of microbiome profiling, metabolomic analyses, microbiota-transfer experiments, and microbiota-targeted interventions.

Human studies ranged from small case reports and exploratory interventions to larger case-control, longitudinal, and population-based cohorts. Preclinical investigations primarily used germ-free animals, chronic-stress paradigms, antibiotic-mediated microbiota depletion, vagotomy, probiotic administration, and fecal microbiota transfer. Considerable heterogeneity in diagnostic definitions, medication exposure, dietary assessment, sequencing methods, sample size, and outcome measures limited direct comparison and precluded quantitative pooling.

For interpretative clarity, the evidence was organized into three categories: human observational evidence, preclinical and translational evidence, and human interventional evidence, summarized in Table 1 (Ref. [23,26,30,31,32,33,34,35,36,37,38,39,40,41,42,43]), Table 2 (Ref. [10,16,27,44,45,46,47,48,49,50,51,52,53,54,55,56,57,58,59,60,61,62]), and Table 3 (Ref. [63,64,65,66,67,68,69]), respectively. This organization allows the strength and clinical relevance of the findings to be evaluated without conflating associations observed in patients with effects produced through experimental manipulation.

**Table 1.** **Human observational evidence on microbiota alterations across psychiatric and neurobehavioral conditions**.

Study	Condition	Population	Key taxa/signatures	Microbiota pattern	Main findings
Castro-Nallar et al., 2015 [35]	Schizophrenia	Adults with schizophrenia and matched controls	*Lactobacillus spp.*; *Bifidobacterium pseudocatenulatum*; *Eubacterium hallii*; *Candida dubliniensis*; *Streptococcus*, *Neisseria*, *Haemophilus*	Oropharyngeal dysbiosis with increased lactic-acid bacteria and fungal abundance; altered richness/evenness and metabolic pathways	Distinct taxonomic and functional profiles; enrichment of glutamate/metabolite transport pathways and reduced energy/carbohydrate metabolism
Chen et al., 2020 [38]	MDD	Human	Firmicutes; Bacteroidetes; Actinobacteria; *Clostridium spp.*; *Anaerostipes*; *Streptococcus*; *Blautia*; *Faecalibacterium*; *Roseburia*	Age-specific dysbiosis: decreased Firmicutes and increased Bacteroidetes in younger patients; opposite Bacteroidetes/Actinobacteria pattern in middle age	Age-specific microbial alterations differentiated patients from controls
Chen et al., 2021 [39]	Depression and anxiety symptoms	Human	Ruminococcaceae; *Akkermansia*; Lactobacillales; *Coprococcus*; *Holdemanella*; *Desulfovibrio*; Bacteroidales; Clostridiales; *Blautia*; *Barnesiella*; Coriobacteriaceae	Inflammation-dependent microbiome effects; no direct dysbiosis measure	CRP × microbiome genetic interaction effects associated with depression and anxiety risk
Evans et al., 2017 [33]	Bipolar disorder	Human	Reduced *Faecalibacterium*; *Anaerostipes*; Ruminococcaceae; *Roseburia*; *Parabacteroides*	Moderate global dysbiosis with depletion of butyrate-producing Firmicutes	Microbiome composition associated with bipolar disorder severity
Huang et al., 2018 [32]	MDD	Human	*Faecalibacterium*; *Coprococcus*; *Blautia*; *Dorea*; Lachnospiraceae; Ruminococcaceae; Clostridiaceae	Reduced alpha diversity; Firmicutes depletion; Proteobacteria enrichment	Identification of gut microbiota alterations in MDD
Jiang et al., 2015 [30]	MDD	Human	*Faecalibacterium*; *Alistipes*; *Blautia*; *Oscillibacter*; *Parabacteroides*; *Ruminococcus*; *Prevotella*; Enterobacteriaceae	Pro-inflammatory dysbiosis with reduced SCFA-producing taxa	Differences between MDD subgroups and controls; associations with symptom severity
Jiang et al., 2018 [31]	GAD	Human	*Faecalibacterium*; *Eubacterium rectale*; *Lachnospira*; *Butyricicoccus*; *Sutterella*; *Bacteroides*; *Escherichia-Shigella*; *Fusobacterium*; *Ruminococcus gnavus*	Reduced richness/diversity; depletion of SCFA-producing taxa; enrichment of pro-inflammatory/pathobiont genera	Dysbiosis identified in active and remissive GAD
Jiang et al., 2024 [40]	Subthreshold depression	Human	*Veillonella atypica*; *Faecalibacterium prausnitzii*; *Eubacterium hallii*; *Coprococcus catus*; *Bacteroides ovatus*	Increased diversity with taxonomic differences versus controls	Dietary patterns and microbiota composition associated with subthreshold depression risk
Hu et al., 2023 [41]	MDD	Patients with mild, moderate, or severe MDD and healthy controls	*Bacteroides*; *Ruminococcus*; *Eubacterium*; additional severity-associated microbial markers	Microbial diversity and composition varied according to MDD severity; increased *Bacteroides* in moderate and severe MDD and depletion of *Ruminococcus* and *Eubacterium*, particularly in severe MDD	Gut microbiota alterations were associated with depressive symptom severity, and candidate microbial markers showed potential discriminatory value
Li et al., 2022 [42]	Bipolar disorder, current depressive episode	Human	*Akkermansia muciniphila*; *Citrobacter*; *Phascolarctobacterium*; *Yersinia*; *Enterobacter*; *Flavobacterium*	Reduced alpha diversity and altered composition	Microbiota, serum metabolome, and brain functional connectivity differed between patients and controls
Malan-Muller et al., 2022 [26]	PTSD; comorbid MDD and anxiety	Human	*Mitsuokella*; *Odoribacter*; *Catenibacterium*; *Olsenella*; Bacteroidetes; *Ruminococcus*; *Akkermansia*	Four-genus consortium enriched in PTSD; Bacteroidetes increased in MDD; medication-associated shifts	Microbiome composition associated with PTSD severity; discriminative microbial consortium identified
Shen et al., 2018 [34]	Schizophrenia	Human	*Succinivibrio*; *Megasphaera*; *Collinsella*; *Clostridium*; *Klebsiella*; *Methanobrevibacter*; *Blautia*; *Coprococcus*; *Roseburia*	Proteobacteria/Fusobacteria enrichment; reduced Firmicutes and SCFA-producing genera	Compositional differences and candidate diagnostic microbial biomarkers
Shu et al., 2025 [43]	Depression and Parkinson’s disease	US adults aged 18–80 years, NHANES 2007–2020	Not reported	Dietary Index for Gut Microbiota reflects diet-related modulation rather than direct dysbiosis	Nonlinear associations with Parkinson’s disease and depression; no significant mediating effect
Xu et al., 2020 [23]	Schizophrenia	Human	Actinobacteria; *Eggerthella*; *Megasphaera*; *Akkermansia muciniphila*; *Bifidobacterium adolescentis*; *Lactobacillus gasseri*; *Clostridium perfringens*; *Enterococcus*	Reduced richness; increased dysbiosis index; pro-inflammatory enrichment and protective-taxa depletion	Microbiota, immune, and metabolic markers associated with schizophrenia
Zhao et al., 2024 [36]	Schizophrenia with metabolic syndrome severity	Human	*Senegalimassilia*; Peptostreptococcaceae; *Acetitomaculum*; *Bacteroides*; *Prevotella*; *Alistipes*	Reduced alpha diversity; depleted Firmicutes/Actinobacteriota; enriched Bacteroidota/Proteobacteria	Microbiota-metabolite panels discriminated metabolic syndrome severity
Zhu et al., 2021 [37]	Schizophrenia	Human	*Haemophilus*; *Coprococcus*; *Megasphaera*; *Succinivibrio*; *Desulfovibrio*; *Clostridium*; *Lachnospira*; *Lactobacillus*	Stage-dependent dysbiosis; beta diversity altered in the acute stage	Specific taxa associated with schizophrenia symptom dimensions

MDD, major depressive disorder; GAD, generalized anxiety disorder; PTSD, Post-Traumatic Stress Disorder; CRP, C-reactive protein; SCFA, short-chain fatty acid; NHANES, National Health and Nutrition Examination Survey.

**Table 2.** **Preclinical and translational evidence on microbiota–brain mechanisms**.

Study	Key taxa or pathway	Manipulation
Bravo et al., 2011 [10]	*Lactobacillus rhamnosus* JB-1	Probiotic administration
Clarke et al., 2013 [16]	Hippocampal serotonin, 5-HIAA, BDNF-related pathways	Post-weaning recolonization
Deng et al., 2021 [50]	*Parabacteroides*; *Ruminococcus*; *Akkermansia*; Lachnospiraceae; *Enterorhabdus*	Citalopram, IDO inhibitor, *Parabacteroides distasonis*
Duan et al., 2021 [51]	Prevotellaceae_UCG-003; Lachnospiraceae; Ruminococcaceae; Lactobacillaceae; Eggerthellaceae; Muribaculaceae; Helicobacteraceae	Escitalopram
Hao et al., 2024 [49]	Proteobacteria; *Bacteroides*; *Lactobacillus*; *Akkermansia*	FMT and Xiaoyaosan
Hu et al., 2022 [52]	*Dialister* sp.; *Roseburia* sp.; *Akkermansia muciniphila*; *Bifidobacterium pseudolongum*; *Veillonella* sp.	FMT from healthy Sprague-Dawley rats
Jang et al., 2018 [46]	*Klebsiella oxytoca*; *Escherichia coli*; *Lactobacillus reuteri*; Lactobacillaceae; Proteobacteria	FMT and *L. reuteri*
Kelly et al., 2016 [44]	*Faecalibacterium*; Prevotellaceae; *Prevotella*; *Eggerthella*; *Holdemania*; *Turicibacter*; *Dialister*; *Anaerofilum*; *Bifidobacterium*; *Roseburia*	Human-to-rat FMT
Khan et al., 2025 [27]	*Bacteroides*; *B. acidifaciens*; Clostridiales bacterium; Proteobacteria; Bacteroidetes; Firmicutes	Multistrain probiotic
Knudsen et al., 2021 [53]	*Lachnospira*; Ruminococcaceae; *Coprococcus*	FMT from healthy donors
Li et al., 2019 [54]	*Lactobacillus*; *Akkermansia*; *Bifidobacterium*; Proteobacteria; Verrucomicrobia; *Alistipes*; *Helicobacter*	FMT
Liu et al., 2024 [48]	*Bacteroides*/Bacteroidaceae; *Clostridium*/Clostridiaceae; *Faecalibacterium*; *Roseburia*; *Adlercreutzia*	FMT and *Clostridium butyricum*
Paiva et al., 2024 [55]	*Bacteroides acidifaciens*; *B. dorei*; *Faecalibaculum rodentium*	FOS + GOS; fluoxetine comparator
Rao et al., 2021 [57]	Not reported	FMT
Shen et al., 2024 [58]	Not reported	FMT and SCFA supplementation
Tian et al., 2020 [59]	*Bifidobacterium breve*; *B. pseudolongum*; *Allobaculum*; *Coprococcus*; *Ruminococcus*; *Akkermansia*; *Bacillus*	B. breve CCFM1025
Wei et al., 2024 [60]	*Faecalibacterium*; *Coprococcus*; *Prevotella*; *Bacteroides*; *Shigella*; *Collinsella*; *Alistipes*; *Blautia*; *Roseburia*	Human-to-mouse FMT
Xu et al., 2024 [61]	*Lachnospiraceae*_NK4A136; *Akkermansia*; *Bacteroides*; *Lactobacillus*; *Bifidobacterium*	FMT and AAV-mediated NLRP3 manipulation
Yuan et al., 2021 [47]	*Prevotella*_9; *Lachnospira*; *Sellimonas*; Lactobacillales; *Enterococcus*; *Streptococcus*	Human observation plus animal metabolite supplementation
Zhang et al., 2023 [56]	Muribaculaceae; Lactobacillaceae; *Alistipes*; *Mucispirillum*; *Faecalibaculum*	SCFA supplementation, FMT, pharmacological modulation
Zheng et al., 2016 [45]	Firmicutes; Actinobacteria; Bacteroidetes; Lachnospiraceae; Ruminococcaceae; *Alistipes*; *Faecalibacterium*	Human-to-mouse FMT
Zhong et al., 2024 [62]	*Ligilactobacillus*; *Faecalibacterium*; *Anaerostipes*; *Muribaculum*; *Phascolarctobacterium*; *Akkermansia*	Oral SP@Rh-gel hydrogel

IBD, inflammatory bowel disease; GABA, gamma-aminobutyric acid; 5-HIAA, 5-hydroxyindoleacetic acid; BDNF, brain-derived neurotrophic factor; IDO, indoleamine 2,3-dioxygenase; FMT, fecal microbiota transplantation; FOS, fructooligosaccharides; GOS, galactooligosaccharides; IL, interleukin; NLRP3, NOD-like receptor protein 3; AAV, adeno-associated virus.

**Table 3.** **Human interventional evidence on microbiota-targeted and treatment-related approaches**.

Study	Condition	Intervention	Design	Key taxa	Microbiota effect	Clinical outcome	Evidence appraisal
Chen et al., 2023 [63]	Depression/anxiety with type 2 diabetes	High-fiber dietary intervention	Human; interventional	Lactobacillus; Akkermansia; Bifidobacterium; Bacteroides; Ruminococcus; Blautia	Improved diversity; reduced Firmicutes/Bacteroidota ratio and opportunistic pathogens	Improved glycemic/lipid indices, inflammatory markers, and mood scores	Preliminary; microbiota-specific contribution cannot be isolated
Collyer et al., 2020 [66]	Depression with irritable bowel syndrome	Fecal microbiota transplantation (FMT)	Human; case-level evidence	Not reported	Conceptual restoration of dysbiosis	Improved depressive and gastrointestinal symptoms; antidepressant reduction/discontinuation	Very low certainty; uncontrolled and highly vulnerable to confounding
Doll et al., 2022 [67]	Treatment-resistant MDD in women	Oral frozen-capsule FMT adjunctive to usual care	Human; case report involving two patients	Alistipes; Oscillibacter; Coprococcus; Ruminococcaceae; Lachnospiraceae; Roseburia; Blautia; Faecalibacterium; Flavonifractor; Bacteroides	Partial restoration of diversity/evenness and enterotype shifts	Reduced HAMD and BDI-II scores; gastrointestinal benefit was partial/transient	Very low certainty; very small sample and short follow-up
Hu et al., 2019 [64]	Bipolar depression	Quetiapine monotherapy	Human; pharmacological longitudinal study	Bacteroidetes; Firmicutes; Faecalibacterium; Roseburia; Coprococcus; Ruminococcus; Parabacteroides; Bacteroides	Reduced diversity and butyrate producers at baseline; treatment-related changes	Microbial features associated with diagnosis and treatment response	Low certainty; medication effects and illness effects are difficult to separate
Parker et al., 2022 [65]	Treatment-resistant bipolar II disorder	FMT via colonoscopy and repeated enemas	Human; single case	Not reported	No direct microbiota profiling reported	Sustained improvement in depressive/hypomanic symptoms, anxiety, sleep, and functioning; medication discontinuation	Very low certainty; single uncontrolled case
Tian et al., 2022 [68]	MDD	B. breve CCFM1025 probiotic	Human; placebo-controlled intervention	Bifidobacterium; Blautia; Bacteroides; Faecalibacterium; Desulfovibrio; Faecalibaculum	Mild compositional changes during treatment	Reduced depressive and gastrointestinal symptoms versus placebo	Low-to-moderate preliminary certainty; strain-specific and limited generalizability
Zhu et al., 2023 [69]	Test-anxiety-related anxiety/depression	Lactobacillus plantarum JYLP-326 for 3 weeks	Human; probiotic intervention	Bacteroides; Roseburia; Faecalibacterium; Prevotella; Bifidobacterium	Stress-related shift partially normalized	Improved psychometric scores with microbiota and fecal-metabolomic changes	Low certainty; short duration and context-specific population

Abbreviations: BDI-II, Beck Depression Inventory-II; FMT, fecal microbiota transplantation; HAMD, Hamilton Depression Rating Scale; MDD, major depressive disorder.

Human observational studies reporting disorder-specific microbiota alterations are summarized in Table 1.

Mechanistic findings derived from germ-free models, microbiota-transfer experiments, probiotic administration, and other controlled manipulations are summarized in Table 2.

Human intervention studies involving dietary modulation, probiotics, pharmacological treatment, and FMT are summarized in Table 3, together with a qualitative appraisal of the current level of evidence.

### 3.3 Gut Microbiota Alterations Across Psychiatric Conditions

Multiple studies reported alterations in microbiota composition in psychiatric patients compared to healthy controls. Changes in microbial diversity were reported along with alterations in the abundance of specific bacterial taxa, especially in bacterial genera involved in metabolic and immune regulation, including *Bifidobacterium*, *Lactobacillus*, *Akkermansia*, *Prevotella*, and *Bacteroides* [23,30,31,32,33,34,35,45,47,70]. At the phylum level, several studies reported alterations involving Firmicutes, Bacteroidetes, Actinobacteria, Proteobacteria, and Verrucomicrobia. However, the direction and magnitude of these changes varied across diagnostic groups, clinical populations, and analytical methods. Table 4 therefore summarizes representative disorder-specific patterns while explicitly indicating conflicting or inconsistent findings rather than assigning a uniform direction of change to each phylum. Alterations in microbial products such as lipopolysaccharide (LPS) have also been implicated as potential links between dysbiosis, barrier dysfunction, and systemic inflammation [12,64,65].

**Table 4.** **Representative microbiota findings at phylum, family, and genus levels across psychiatric disorders and their reported heterogeneity**.

Psychiatric condition	Phylum or taxonomic group	Representative findings	Direction and consistency	Interpretative considerations
Major depressive disorder	Firmicutes (Bacillota)	Several studies reported reduced abundance of Firmicutes and depletion of butyrate-producing families or genera, including members of Lachnospiraceae and Ruminococcaceae.	Frequently decreased, but not uniformly across studies	Findings may depend on age, diet, medication exposure, disease severity, and microbiota-profiling methods.
Major depressive disorder	Bacteroidetes (Bacteroidota)	Increased abundance was reported in some cohorts, whereas other studies found reductions or no significant differences.	Conflicting	Phylum-level findings may conceal divergent genus- and species-level patterns.
Major depressive disorder	Proteobacteria (Pseudomonadota)	Enrichment of potentially pro-inflammatory taxa was reported in some studies.	Often increased, but inconsistent	Associations may reflect inflammation, gastrointestinal comorbidity, medication exposure, or metabolic status.
Bipolar disorder	Firmicutes (Bacillota)	Reduced abundance of Faecalibacterium and other butyrate-producing taxa was reported in several studies.	Tendency toward depletion of selected taxa; phylum-level direction inconsistent	Findings vary according to mood state, treatment exposure, and metabolic comorbidity.
Bipolar disorder	Bacteroidetes (Bacteroidota)	Some studies reported enrichment, particularly during depressive episodes, whereas others identified different compositional profiles.	Heterogeneous	No reproducible phylum-level signature has been established.
Anxiety disorders	Firmicutes (Bacillota)	Reduced abundance of short-chain-fatty-acid-producing genera was reported in some cohorts with generalized anxiety disorder.	Often decreased at selected taxonomic levels	Evidence remains limited and primarily cross-sectional.
Anxiety disorders	Bacteroidetes (Bacteroidota) and Proteobacteria (Pseudomonadota)	Altered abundance of Bacteroides, Escherichia–Shigella, and other potentially pro-inflammatory taxa was described.	Variable	Findings differ between active and remitted symptoms and may be influenced by gastrointestinal status.
Schizophrenia	Firmicutes (Bacillota)	Some studies reported reduced Firmicutes and depletion of short-chain-fatty-acid-producing taxa.	Frequently decreased in selected cohorts, but inconsistent	Antipsychotic exposure, smoking, diet, obesity, and illness duration are major confounders.
Schizophrenia	Bacteroidetes (Bacteroidota), Actinobacteria (Actinomycetota), and Proteobacteria (Pseudomonadota)	Enrichment or depletion of these phyla varied across studies.	Conflicting and stage-dependent	No phylum-level alteration is sufficiently reproducible for diagnostic use.
Post-traumatic stress disorder	Bacteroidetes (Bacteroidota) and Firmicutes (Bacillota)	Limited human studies reported compositional differences involving Bacteroidetes, Ruminococcus, Akkermansia, and related taxa.	Preliminary and inconsistent	Evidence is based on small samples and is sensitive to trauma exposure, medication, diet, and comorbidity.
Transdiagnostic findings	Verrucomicrobia (Verrucomicrobiota), Fusobacteria (Fusobacteriota), and other less frequently reported phyla	Alterations were reported sporadically across mood, anxiety, and psychotic disorders.	Insufficiently replicated	These findings should be regarded as exploratory and require validation in independent cohorts.

Note: Phylum-level findings should be interpreted cautiously because broad taxonomic categories may conceal opposing genus- or species-level changes. The direction of alteration is not uniform across disorders or study populations. Legacy taxonomic names used in many of the considered studies are reported first, followed by updated nomenclature in parentheses.

Studies of mood and anxiety disorders frequently reported alterations in alpha and beta diversity, but the direction and magnitude of these changes were not uniform. Findings in schizophrenia were even more heterogeneous, with reports of reduced, increased, or unchanged diversity depending on the population, illness stage, medication exposure, and analytical approach. Accordingly, neither diversity measures nor broad phylum-level patterns currently provide a reproducible disorder-specific microbial signature [30,31,32,33,34,35,36,37,44,45,47,70,71].

Among the studies reporting these alterations, some provided particularly detailed characterizations of microbial shifts. For example, Jiang et al. [30] found significant shifts in gut microbial composition in patients with major depressive disorder, including reduced diversity and depletion of short-chain-fatty-acid-producing taxa compared with healthy controls. Similarly, in generalized anxiety disorder (GAD), they observed reduced microbial richness, depletion of potentially beneficial genera, and enrichment of potentially pro-inflammatory taxa [31].

The more limited evidence concerning Post-Traumatic Stress Disorder (PTSD) indicated that specific genera were found to differentiate PTSD patients from trauma-exposed controls [26], while preclinical stress models showed that microbiota alterations can induce PTSD-like behaviors [27].

Studies focusing on schizophrenia have reported heterogeneous findings regarding gut microbiota diversity and composition. Shen et al. [34] identified significant compositional differences between patients with schizophrenia and healthy controls and explored the potential diagnostic value of microbiota-based signatures. Investigations of the oropharyngeal microbiome identified additional taxonomic and functional differences with potential biomarker relevance [35]. Other studies reported associations with inflammatory, metabolic, and symptom-related characteristics, although no reproducible diagnostic signature has been established [23,36,37].

### 3.4 Biological Pathways Linking Gut Microbiota and Brain Function

Across the included studies, microbiota–brain communication converged on several interconnected biological domains: microbial metabolite production, immune-inflammatory signaling, neuroendocrine regulation, neurotransmitter-related pathways, neural communication through the vagus nerve, and intestinal and blood-brain barrier integrity.

Altered short-chain fatty acid production, tryptophan-kynurenine metabolism, and other microbial metabolic pathways were recurrently implicated in the modulation of immune activity, epithelial integrity, and neural signaling. Dysbiosis was also associated with increased systemic and intestinal inflammation, including alterations in interleukin-6, interleukin-1β, tumor necrosis factor-α, Toll-like receptor 4/nuclear factor-κB signaling, and NLRP3 inflammasome activation [39,41,48,53,55,64,65]. These mechanisms may contribute to impaired barrier function and altered neuroimmune communication, although direct mediation has not been established consistently in human psychiatric populations.

Neuroendocrine and neurotransmitter-related pathways included HPA axis regulation, serotonergic, GABAergic, dopaminergic, and glutamatergic signaling, and vagal afferent communication. Foundational germ-free and vagotomy studies established early mechanistic plausibility for these pathways, whereas subsequent experimental research expanded the evidence to include fecal microbiota transfer, probiotic administration, and modulation of microbial metabolites [10,16,39,48,50,54,57,64,65].

The strength of inference differed substantially across study types. Controlled animal experiments demonstrated that microbiota manipulation can alter behavioral, immune, neurochemical, and neuroendocrine outcomes. In contrast, human studies primarily identified associations between microbial profiles, inflammatory status, symptom severity, and treatment response. Accordingly, the literature supports a multisystem model of microbiota-brain communication rather than a single linear causal pathway.

## 4. Discussion

### 4.1 Human and Preclinical Evidence: Different Levels of Inference

The evidentiary status of microbiota-brain findings differs substantially between human and animal research. In humans, most available studies are cross-sectional and identify associations between microbiota profiles and psychiatric diagnosis, symptom severity, inflammatory status, cognition, metabolic characteristics, or treatment exposure. These designs cannot establish temporal directionality and are susceptible to residual confounding from diet, body mass index, psychotropic medication, antibiotic or probiotic use, smoking, alcohol consumption, gastrointestinal disorders, physical activity, sleep, and socioeconomic factors. Consequently, no disorder-specific microbial signature can currently be considered causally established or sufficiently reproducible for routine clinical use.

Human longitudinal and interventional evidence remains more limited. Although some studies have reported microbiota changes following pharmacological treatment, dietary intervention, probiotic supplementation, or FMT, sample sizes are generally small, follow-up periods are short, and intervention protocols are heterogeneous. Moreover, improvements in psychiatric symptoms do not necessarily demonstrate that microbiota modification was the principal mechanism of benefit [43,59,60,61,62,66,67].

By contrast, preclinical studies provide greater experimental control. Germ-free models, antibiotic-mediated depletion, vagotomy experiments, probiotic administration, and fecal microbiota transfer allow direct manipulation of microbial exposure and have demonstrated effects on stress reactivity, neurotransmitter signaling, immune activation, intestinal permeability, neuroendocrine function, and behavioral outcomes. These findings provide mechanistic plausibility and support the possibility that the microbiota can contribute to brain-related processes under controlled conditions [10,16,27,38,39,40,41,42,48,49,50,51,52,53,54,55,56,57,58,63,64,65].

Nevertheless, preclinical findings should not be directly equated with established mechanisms in patients. Rodent behavioral measures model selected dimensions such as anhedonia-like behavior, avoidance, stress reactivity, or social interaction, but they do not reproduce the full phenomenology of depression, bipolar disorder, schizophrenia, anxiety disorders, or post-traumatic stress disorder [12,27,38,39,40,41,42,48,49,50,51,52,53,54,55,56,57,58,63,64,65]. The strongest interpretation supported by the current literature is therefore that human studies demonstrate clinically relevant associations, whereas animal studies provide experimental evidence of potential mechanisms whose translational relevance still requires confirmation. The early preclinical studies retained in this review are interpreted as foundational evidence that established the experimental plausibility of microbiota-dependent neural, neuroendocrine, and behavioral effects. Their specific findings are described in the mechanistic sections above and are not reiterated here. Importantly, these studies represent historical milestones in experimental research rather than direct evidence of equivalent causal mechanisms in psychiatric patients [10,12,16].

### 4.2 Summary of Main Findings

The principal finding of this review is that microbiota-related alterations across psychiatric disorders converge more consistently at the level of biological function than at the level of individual microbial taxa. Recurrent themes included reduced short-chain-fatty-acid-producing capacity, altered tryptophan metabolism, immune-inflammatory activation, neuroendocrine dysregulation, impaired barrier integrity, and altered neural signaling [12,13,14,15,16,31,32,39,48,54,55,64,65]. Taxonomic findings were heterogeneous and no disorder-specific microbial signature was sufficiently reproducible for clinical application.

Findings suggest that the gut microbiota may contribute to the modulation of brain function and behavior through multiple interacting biological pathways; however, causal inference in humans remains limited [12]. In humans, most available studies are cross-sectional and demonstrate associations between microbiota profiles, symptom burden, inflammatory markers, or treatment status. These designs cannot establish directionality and remain vulnerable to confounding by diet, medication, body mass index, smoking, gastrointestinal comorbidity, and lifestyle. In contrast, germ-free models, antibiotic depletion, vagotomy experiments, and microbiota-transfer paradigms permit controlled manipulation and provide stronger mechanistic evidence that microbial changes can alter neuroimmune, neuroendocrine, and behavioral outcomes. Nevertheless, animal behavioral phenotypes are imperfect proxies for complex psychiatric disorders, and these findings should not be interpreted as direct demonstrations of causality in patients.

### 4.3 Biological Pathways in Gut-Brain Interactions

One of the most implicated pathways involved in gut-brain interactions is neurotransmitter signaling, since gut microbes produce neuroactive molecules such as serotonin, GABA, dopamine and glutamate [7,12]. These neurotransmitters do not directly influence the CNS since they cannot cross the blood-brain barrier, instead they use different pathways such as vagal afferents, immune mediators, and metabolic signaling [11,12]. In this context, vagal signaling has been proposed as a critical neural pathway mediating microbiota-brain communication, as preclinical studies demonstrated that after subdiaphragmatic vagotomy some of the behavioral and neurochemical effects vanished [10]. Immune activation and neuroinflammation are considered key mechanisms as well, since over production of pro-inflammatory cytokines leads to an increase in intestinal permeability allowing microbial products translocation [70,71]. Besides, gut microbiota can influence the HPA axis modulating stress reactivity, since preclinical studies showed that germ-free animals display altered stress-related neurobiological responses [16].

Neurotransmitter modulation, immune signaling, endocrine regulation, and vagal pathways form an integrated network through which gut microbiota may influence brain function [12].

### 4.4 Microbiota Alterations in Neuropsychiatric Disorders

According to the reviewed literature psychiatric disorders seem to share microbiota-related mechanisms. In bipolar disorder and MDD, alterations in gut microbiota composition have been associated with clinical characteristics, including symptom severity, in observational human studies [35,37,52,58]. In isolated uncontrolled human case reports and case series involving patients with bipolar disorder, MDD, or depression associated with irritable bowel syndrome, FMT has been associated with improvements in depressive symptoms. These preliminary observations do not establish efficacy because of the small samples, absence of control groups, and potential confounding by concomitant treatments [64,66,67]. Dysbiosis has also been linked to anxiety disorders with the involvement of serotonergic and GABAergic signaling [22]. Dysbiosis has also been associated with anxiety disorders, potentially involving serotonergic, GABAergic, immune-inflammatory, and vagal pathways [7,10,12,22,32]. Experimental studies further showed that microbiota depletion or transfer can induce or reverse anxiety-like phenotypes and associated changes in intestinal permeability, inflammation, and neurochemical signaling [39,41,52,65].

A clinical study reported alterations in microbiota profiles of individuals with PTSD compared to trauma‑exposed controls. In a cohort of PTSD patients, it was found that genera including Mitsuokella, Olsenella and Oligosphaera were significantly associated with PTSD status and with symptom severity, emerging as potential markers distinguishing PTSD cases from trauma‑exposed individuals [26]. In preclinical models, gut dysbiosis induced by prolonged stress was associated with PTSD‑like behaviors [27].

Studies of schizophrenia have reported alterations in gut and oropharyngeal microbial composition, although findings concerning diversity and individual taxa remain heterogeneous and are influenced by illness stage, medication exposure, smoking, diet, obesity, and metabolic comorbidity [23,36,37,44,70]. Several cohorts have described depletion of selected short-chain-fatty-acid-producing taxa and enrichment of microorganisms associated with inflammatory or metabolic dysregulation, but no reproducible microbial signature is currently suitable for diagnostic use [23,36,37,44,70].

Immune-inflammatory mechanisms may represent one pathway linking microbiota alterations with schizophrenia-related neurobiology. Impaired intestinal barrier integrity and increased exposure to microbial products may contribute to peripheral immune activation and altered cytokine signaling. These processes have been hypothesized to interact with microglial activation, oxidative stress, and neurodevelopmental or synaptic abnormalities. Nevertheless, direct mediation between specific microbial changes, immune activation, and psychotic symptoms has not been established in clinical populations [23,24,36,37,70].

Metabolomic investigations have identified differences involving short-chain fatty acids, tryptophan–kynurenine metabolism, bile acids, amino-acid metabolism, and lipid pathways. Such findings support the value of integrating taxonomic and functional analyses, but the reported metabolite profiles are not specific to schizophrenia and may partly reflect dietary patterns, smoking, metabolic syndrome, illness duration, or treatment exposure. Experimental transfer of microbiota from patients with schizophrenia to rodents has produced behavioral, metabolic, and brain-transcriptional alterations, providing mechanistic plausibility while not constituting direct evidence of causality in humans.

Antipsychotic treatment is both an important confounder and a potential modifier of microbiota–host interactions. Antipsychotic drugs may influence microbial composition indirectly through changes in appetite, body weight, glucose metabolism, gastrointestinal motility, and dietary behavior, and possibly through direct antimicrobial or metabolic effects. Conversely, interindividual microbial differences may contribute to variation in drug metabolism and treatment-associated metabolic adverse effects. Future studies should therefore prioritize medication-naïve or first-episode populations, longitudinal sampling before and after treatment initiation, and careful assessment of metabolic and lifestyle variables.

### 4.5 Therapeutic Implications and Emerging Targets

Randomized placebo-controlled trials of probiotics in patients with depressive symptoms have reported mixed findings, with symptom improvement in some studies and no significant benefit over placebo in others [64,72]. However, the clinical maturity and certainty of evidence differ substantially across these approaches.

Probiotics and psychobiotics currently represent the most extensively investigated microbiota-targeted interventions in psychiatric populations, although their proposed effects remain strain-specific and may involve interacting neural, immune, endocrine, and metabolic pathways [68]. Randomized and placebo-controlled trials have reported reductions in depressive or anxiety symptoms in some populations, although null findings have also been reported and the magnitude of benefit remains inconsistent across studies [28,66,67,69]. Comparability is limited by differences in bacterial strains, single-strain versus multistrain formulations, dosage, treatment duration, baseline microbiota composition, diagnostic criteria, symptom severity, concomitant psychotropic treatment, and outcome measures [28,68,73]. Moreover, several studies have small samples and short follow-up periods, and improvements in symptom-rating scales are not always accompanied by consistent inflammatory, metabolic, or microbiome changes. Current findings therefore support a possible adjunctive effect rather than established antidepressant or anxiolytic efficacy.

Evidence concerning prebiotics is more limited. Most available studies are preclinical or involve small human samples, and many assess surrogate outcomes such as microbial diversity, short-chain fatty acid production, inflammatory markers, or stress-related biomarkers rather than remission, relapse prevention, functional recovery, or other clinically meaningful psychiatric endpoints. Consequently, the clinical significance of prebiotic-associated microbiota changes remains uncertain.

Dietary interventions, particularly fiber-rich, Mediterranean-style, or microbiota-supportive dietary patterns, may improve mood symptoms and metabolic health while promoting microbial diversity and short-chain fatty acid production [12,43,74]. Nevertheless, these interventions affect multiple biological and behavioral pathways simultaneously, including body weight, insulin sensitivity, inflammation, nutrient availability, sleep, and lifestyle regularity. It is therefore difficult to determine what proportion of the observed psychiatric benefit is specifically mediated by microbiota modification. Dietary strategies are clinically attractive because of their broader health benefits and relatively favorable safety profile, but microbiota-mediated efficacy should not be inferred from symptomatic improvement alone.

FMT provides a more direct means of altering the gut microbial ecosystem. Preclinical transfer studies offer strong mechanistic plausibility by demonstrating that microbiota manipulation can modify behavioral, immune, metabolic, and neuroendocrine outcomes under controlled experimental conditions. In psychiatric patients, however, the clinical literature remains limited mainly to case reports, small case series, and early exploratory studies [59,60,62]. Reported improvements in mood, anxiety, sleep, or functioning must therefore be interpreted cautiously because of the absence of adequate control groups, small samples, expectancy effects, concomitant treatment changes, variable donor-selection procedures, heterogeneous administration protocols, and limited long-term follow-up. Safety concerns, including transmission of infectious agents, unintended metabolic or immune effects, and uncertain durability of microbiota engraftment, further preclude routine psychiatric use.

The certainty of evidence for microbiota-targeted interventions in psychiatric disorders remains low. Probiotic trials provide the largest body of human interventional evidence, but their findings remain inconsistent. Some studies report modest improvements in selected symptom scales, whereas others show no significant advantage over placebo. The available evidence therefore does not support a uniform class effect, and clinical interpretation must remain strain-specific, population-specific, and outcome-specific.

Prebiotic evidence is preliminary, dietary studies cannot isolate microbiota-specific mechanisms, and FMT has stronger preclinical than clinical support. Current evidence does not justify the use of these approaches as stand-alone treatments or as substitutes for established pharmacological and psychotherapeutic interventions. At present, they should be regarded as investigational or adjunctive strategies whose potential value may depend on patient characteristics, baseline microbial profiles, metabolic and inflammatory status, diet, medication exposure, and psychiatric diagnosis.

The translational trajectory of *Lactobacillus rhamnosus* JB-1 illustrates the gap between promising preclinical findings and human clinical outcomes. Although this strain produced vagus-dependent behavioral, neuroendocrine, and GABAergic effects in mice [10], an eight-week randomized, placebo-controlled crossover study in healthy men found no significant effects on mood, anxiety, perceived stress, sleep quality, hypothalamic–pituitary–adrenal axis reactivity, inflammatory markers, cognition, or electroencephalographic measures [75]. These findings indicate that strain-specific effects observed in selected animal models cannot be assumed to translate directly to humans.

Negative or inconclusive findings have also been reported with other probiotic formulations. In an eight-week double-blind, randomized, placebo-controlled trial involving 79 participants selected for low mood, supplementation with *Lactobacillus helveticus* and *Bifidobacterium longum* did not significantly improve psychological outcomes compared with placebo [72]. Meta-analytic evidence is similarly mixed: some analyses have reported modest benefits for selected depressive or anxiety measures, whereas others have found no significant effect on anxiety or no improvement across several commonly used symptom scales [76]. These inconsistencies may reflect differences in strain specificity, host characteristics, baseline microbiota, diagnostic status, concomitant treatments, treatment duration, and outcome selection. Accordingly, probiotics should not be treated as a homogeneous therapeutic class, and positive findings obtained with one strain or population cannot be generalized to other formulations or psychiatric conditions [75,77].

Future trials should use adequately powered randomized designs, standardized strains or intervention protocols, predefined clinically meaningful outcomes, longer follow-up periods, and systematic assessment of adverse events. Integration of microbiome profiling with metabolomic, inflammatory, neuroendocrine, and clinical measures may help identify biological responders and clarify whether observed symptom changes are actually mediated by microbiota modification. Until such evidence is available, personalized microbiota-based treatment remains a promising research direction rather than an established component of routine psychiatric care.

### 4.6 Limitations

Several limitations should be considered when interpreting the evidence summarized in this review. A large proportion of available human studies are cross-sectional or observational in design. As a result, they can identify associations between microbiota composition and psychiatric phenotypes but cannot establish directionality or causality. It remains unclear whether microbiota alterations contribute to the onset or persistence of psychiatric symptoms, represent downstream consequences of illness-related behaviors, or reflect the effects of diet, medication, lifestyle, comorbid medical conditions, or environmental exposures.

Microbiota profiling methods are highly heterogeneous across studies. Differences in sample collection, storage conditions, DNA extraction protocols, sequencing platforms, bioinformatic pipelines, taxonomic resolution, and statistical approaches limit comparability and reproducibility. In addition, many studies report taxonomic changes without integrating functional metagenomics, metabolomics, immune profiling, or neuroendocrine markers, making it difficult to infer the biological relevance of specific microbial signatures.

The translatability of rodent models to human psychiatric disorders remains limited. Preclinical studies provide valuable mechanistic insight and allow experimental manipulation of microbiota composition, FMT, antibiotic exposure, or probiotic administration. However, animal models only approximate selected behavioral or neurobiological dimensions of complex psychiatric disorders and cannot fully reproduce the subjective, developmental, social, and clinical complexity of human psychopathology.

Confounding factors are not consistently controlled across clinical studies. Diet, body mass index, psychotropic medication, antibiotics, probiotics, gastrointestinal symptoms, smoking, alcohol use, sleep patterns, physical activity, socioeconomic status, and comorbid inflammatory or metabolic conditions may all influence microbiota composition and psychiatric outcomes. The absence of standardized confounder control reduces the specificity of reported associations and may partly explain inconsistencies across studies. Another major gap is the limited integration of host genomic and epigenomic information, which restricts understanding of how genetic susceptibility and host-microbiota interactions contribute to variability across psychiatric phenotypes and treatment outcomes.

Publication bias should be considered. Studies reporting significant microbiota differences or positive effects of microbiota-targeted interventions may be more likely to be published than negative or inconclusive findings. This may lead to an overestimation of the strength and consistency of the association between gut microbiota dysbiosis and psychiatric symptoms. The literature search was limited to PubMed, which may have reduced the breadth of study identification. Relevant publications indexed exclusively in other databases, including Scopus, Web of Science, Embase, or PsycINFO, may therefore not have been captured. A formal study-level risk-of-bias assessment and quantitative certainty-of-evidence grading were not performed. Accordingly, the review cannot provide numerical proportions of studies classified as high, moderate, or low quality, and its conclusions should be interpreted as a critical narrative synthesis rather than as a formally graded systematic evidence assessment.

Finally, although microbiota-targeted interventions such as probiotics, prebiotics, dietary modulation, and FMT are promising, current evidence remains preliminary. Many intervention studies are limited by small sample sizes, short follow-up periods, heterogeneous formulations or protocols, and variable psychiatric outcome measures. Therefore, these approaches should currently be considered adjunctive and investigational rather than established stand-alone treatments for psychiatric disorders.

## 5. Conclusions

The gut-brain axis is supported by substantial mechanistic evidence from controlled preclinical models, including germ-free experiments, vagotomy studies, microbial metabolite manipulation, probiotic administration, and fecal microbiota transfer. These models demonstrate that microbiota-related interventions can modify neurochemical, immune, neuroendocrine, barrier-related, and behavioral outcomes under controlled conditions. By contrast, human evidence remains predominantly observational and supports associations between microbial profiles, psychiatric symptoms, inflammatory status, and treatment response, without yet establishing directionality or causality. These associations appear to involve multiple, partially overlapping mechanisms, including changes in microbial metabolite production, tryptophan and short-chain fatty acid metabolism, immune-inflammatory activation, HPA axis dysregulation, neurotransmitter signaling, intestinal and blood-brain barrier integrity, and vagus nerve-mediated communication.

Although human studies remain predominantly observational and heterogeneous, controlled preclinical experiments provide stronger mechanistic evidence that microbiota manipulation can affect behavioral, neurochemical, immune, and neuroendocrine outcomes. These experimental findings should not, however, be interpreted as direct evidence of causality in human psychiatric disorders. These findings suggest that microbiota dysbiosis should not be viewed merely as an epiphenomenon of psychiatric illness, but rather as a potential modulator of disease vulnerability, symptom severity, and treatment response. At the same time, current evidence does not yet justify the use of microbiota-targeted interventions as stand-alone treatments in psychiatry. Probiotics, dietary modulation, prebiotics, and FMT should instead be considered promising adjunctive strategies whose clinical application requires further validation. Across the included literature, consistency was greater for broad functional pathways than for individual microbial taxa. Recurrent findings included reduced representation of short-chain-fatty-acid-producing bacteria, enrichment of potentially pro-inflammatory taxa, altered tryptophan-kynurenine metabolism, immune activation, impaired barrier integrity, and dysregulation of neuroendocrine signaling. However, the direction and magnitude of taxonomic alterations varied across studies, and no microbial signature was consistently specific to a single psychiatric disorder. The most robust mechanistic support derived from controlled animal experiments, whereas human studies predominantly demonstrated associations. Major evidence gaps included the limited number of longitudinal and adequately powered intervention studies, insufficient replication across independent cohorts, poor control of dietary and medication-related confounders, and lack of standardized microbiome sampling, sequencing, and analytical procedures.

Research should prioritize longitudinal, mechanistically informed, and adequately powered clinical studies integrating microbiome profiling with metabolomic, inflammatory, neuroendocrine, neuroimaging, and host-genomic data. Particular attention should be given to host genetic variation and gene-microbiota interactions, because genetic differences may influence microbial colonization, immune responsiveness, intestinal barrier function, metabolite production, and susceptibility to psychiatric symptoms. Conversely, microbial metabolites and inflammatory signals may modify host gene expression and epigenetic regulation, creating bidirectional host-microbiota effects that may partly explain interindividual variability in disease risk, symptom expression, and treatment response.

Future studies should therefore combine genomic, epigenomic, metagenomic, metabolomic, and clinical data within longitudinal designs and should examine whether specific host-microbiota profiles identify biologically meaningful patient subgroups. Standardization of microbiota assessment methods, intervention protocols, donor-selection criteria for fecal microbiota transplantation, and psychiatric outcome measures will also be essential to improve reproducibility and clinical translation. A more precise characterization of host genetic background, environmental exposures, medication use, diet, and microbial function may ultimately support the development of stratified and personalized microbiota-informed approaches for prevention, symptom modulation, and augmentation of established psychiatric treatments.

## Abbreviations

ENS, enteric nervous system; CNS, central nervous system; HPA, hypothalamic-pituitary-adrenal; GABA, γ-aminobutyric acid; SERT, serotonin transporter; SCFAs, short-chain fatty acids; FMT, fecal microbiota transplantation; MDD, major depressive disorder; GAD, generalized anxiety disorder; PTSD, post-traumatic stress disorder; LPS, lipopolysaccharide; BDNF, brain-derived neurotrophic factor; CRP, C-reactive protein.

## Declaration of AI and AI-Assisted Technologies in the Writing Process

During manuscript preparation, the authors used Google Gemini 2.0, accessed in May 2026, to support the generation of illustrative figures under the authors’ guidance and direction. ChatGPT by OpenAI was used to assist with English-language editing and to improve the clarity, grammar, and readability of selected passages. These tools were not used to generate or interpret scientific data, perform the literature search, select studies, assess evidence, or formulate the scientific conclusions. All AI-assisted outputs were critically reviewed and edited by the authors, who take full responsibility for the accuracy, integrity, and final content of the manuscript.
